# My Lovely Granny’s Farm: An immersive virtual reality training system for children with autism spectrum disorder

**DOI:** 10.1007/s10639-023-11862-x

**Published:** 2023-05-20

**Authors:** Aiganym Soltiyeva, Wilk Oliveira, Alimanova Madina, Shyngys Adilkhan, Marat Urmanov, Juho Hamari

**Affiliations:** 1 Faculty of Engineering and Natural Sciences, Suleyman Demirel University, Kaskelen, Kazakhstan; 2 Gamification Group, Faculty of Information Technology and Communication Sciences, Tampere University, Tampere, Finland

**Keywords:** Autism spectrum disorder, Virtual reality, Immersive systems, Social interaction, Communicational skills

## Abstract

One of the biggest difficulties faced by children with Autism Spectrum Disorder during their learning process and general life, is communication and social interaction. In recent years, researchers and practitioners have invested in different approaches to improving aspects of their communication and learning. However, there is still no consolidated approach and the community is still looking for new approaches that can meet this need. Addressing this challenge, in this article we propose a novelty approach (i.e., an Adaptive Immersive Virtual Reality Training System), aiming to enrich social interaction and communication skills for children with Autism Spectrum Disorder. In this adaptive system (called My Lovely Granny’s Farm), the behavior of the virtual trainer changes depending on the mood and actions of the users (i.e., patients/learners). Additionally, we conducted an initial observational study by monitoring the behavior of children with autism in a virtual environment. In the initial study, the system was offered to users with a high degree of interactivity so that they might practice various social situations in a safe and controlled environment. The results demonstrate that the use of the system can allow patients who needed treatment to receive therapy without leaving home. Our approach is the first experience of treating children with autism in Kazakhstan and can contribute to improving the communication and social interaction of children with Autism Spectrum Disorder. We contribute to the community of educational technologies and mental health by providing a system that can improve communication among children with autism and providing insights on how to design this kind of system.

## Introduction

Children with Autism Spectrum Disorder (ASD) are defined as people who have trouble with communication and adapting to society (Autism Research Institute, 2021). One in 54 children in the USA is diagnosed with ASD (Autism Research Institute, 2021; Autism Speaks, 2021), and the situation is similar in many countries around the world (Autism Speaks, 2021). For example, according to the Ministry of Education and Science, 6771 children with autism live in Kazakhstan (Forbes Kazakhstan, 2021). Deepening this problem, families with autistic children face serious obstacles such as a lack of information, a lack of specialist treatment, and prejudiced attitudes from society (Forbes Kazakhstan, 2021).

In the last few years, alternative ways (e.g., digital games, gamification, virtual reality (VR)) have been proposed to address this problem in different countries (Finkelstein et al., 2013; Rahmadiva et al., 2019; Lele, 2013). Among the emerging technologies, training systems are helpful for the psychological therapy of children with ASD who would not have access to the required medical care (Ramachandiran et al., 2015). At the same time, Immersive Virtual Reality Systems (IVRS) allow people to completely immerse themselves into a virtual world created on the computer (Hocking et al., 2022, Radianti et al., 2020). This occurs because the effects created in VR environments are projected onto the human mind and allow people to have feelings that are as close to real feelings as possible (Alcañiz et al., 2022; Radianti et al., 2020). Thus, users are able to train and face their traits in a safe and controlled environment (Hocking et al., 2022). Using IVRS for training has shown high effectiveness because children with ASD had a better perception of visual information (Ramachandiran et al., 2015; Halabi et al., 2017). Earlier research has also found that applications with VR have the potential to provide effective and innovative clinical treatments for individuals with autism (Autism Research Institute, 2021; Alcañiz et al., 2022; Arthur et al., 2021; Miller et al., 2020).

Aiming to face the challenge of improving the communication and social skills of children with ASD, we propose an adaptive IVRS, specifically developed to intensify the social interaction and communication abilities of children with autism. Advanced new VR headsets can recognize the user’s facial expressions, which helps to analyze and evaluate the emotional state of the participant. Children with ASD have difficulty facing new surroundings and carrying out tasks to train adaptations to new situations. The major significance of this IVRS is that users could start the training lessons again, and also repeat activities to practice skills in an enjoyable environment.

This study focuses on the following objectives: *i)* create realistic virtual environments for the behavioral training process of children with autism, *ii)* develop face and speech recognition algorithms and apply emotions to a virtual avatar in real-time according to the behavior of the patient, *ii)* explore with professional psychotherapists if the system meets the requirements of therapy. We co-created two levels with different tasks for training children. To explore the system quality, we conducted a study with 12 children (4–15 years old) with an ASD diagnosis. The duration of the training was three months. All of the processes of the training have been recorded for data collection and analysis. The main results indicate that using VR for rehabilitation has positive effects on improving the communication skills of children with ASD. The study contributes to the communities of educational technologies, mental health, and social communication, by proposing a system to improve the communication and social interaction of children with ASD, and providing insights on how to design IVRS for autistic children.

## Background

In this section, we present an overview of the main topics addressed in this article (Autism and Virtual Reality) and the main related works.

### Autism and virtual reality

Among the typical cases of children with autism, it is possible to identify children with four main behaviors that differ in their systemic characteristics. The first group includes children who do not develop an active selectivity in their contact with the environment and people, which is evident in their field behavior. They are practically unresponsive and do not use speech or non-verbal means of communication on their own (Никольская, 2014).

The second group includes children who have only the simplest forms of active contact with people. They develop habitual forms of life, but they are rigidly limited and the child strives to defend their immutability (i.e. the desire to maintain constancy in the environment), and their habitual order of life seen in things such as their selectivity in food, clothing, and routing of walks, is maximally expressed here. These children are suspicious of everything new, fearful of the unexpected, may exhibit pronounced sensory discomfort and squeamishness, easily and rigidly register discomfort and fright, and accordingly may accumulate persistent fears (Никольская, 2014).

Children from the third group have an unfolding but highly indirect form of contact with the outside world and people, demonstrating fairly complex but rigid programs of behavior (including speech), poorly adapted to changing circumstances, and have stereotypical hobbies, often associated with unpleasant acute experiences. This creates extreme difficulties in interacting with people and circumstances, with such issues as the child’s autism manifesting as a preoccupation with their own stereotypical interests and an inability to build dialogical interactions (Никольская, 2014).

For children from the fourth group, an arbitrary organization is very difficult, but in principle accessible. In contact with others, they tire quickly, may become exhausted and overexcited, and have pronounced problems organizing their attention, focusing on and yet fully understanding speech instructions (Almanac Institute of special education, 2022).

Uta Frith Emeritus Professor of Cognitive Neuroscience from University College London, states that concentration and attention to detail are typical characteristics of a person with autism, extremely gifted in some areas (Publishing House PostNauka, 2023). Autism is extremely heterogeneous: every person with autism is different. It’s a particular cognitive style, a cognitive phenotype, that varies from person to person. (Publishing House PostNauka, 2023). Even the earliest descriptions noted that in some cases, children with autism or Asperger’s syndrome show special abilities in one or more areas: mathematics, music, drawing, and others. This phenomenon is called “savantism” (Bal et al., 2022). Savantism usually becomes noticeable in children with autism at the age of 5–10 years old (Тевелев, 2022). There is some suggestion that among famous people savants were the musician Wolfgang Amadeus Mozart, the mathematician Grigory Perelman, and the physicist Albert Einstein. Autistic children with special talents are often well-socialized and move toward society. One prime example is the writer, mathematician, and computer scientist Daniel Tammet. He speaks many languages and has written several books about how people with autism perceive the world (Тевелев, 2022).

Virtual Reality has been used as a training tool since the end of the last century (Radianti et al., 2020). According to early studies, improving social communication skills may lead to improving the daily life of people with autism (Park et al., 2012). Despite the high cost, the first generation of VR equipment has been used for training purposes (Checa & Bustillo, 2020). VR-based simulations are specially used for military applications because they offer the opportunity to conduct exercises in safe and cost-effective environments (Lele 2013; Pallavicini et al., 2016). VR devices are also widely used in medicine, sports, and other manufacturing industries (Justham et al., 2004; Miles et al., 2012; Fuhua et al., 2002; Ruikar et al., 2018).

The feeling of full immersion into the VR environment is the main factor for its use in various fields for training (Mikropoulos & Natsis, 2011). Furthermore, two other key factors have influenced the large-scale development of VR. The first is the cost reduction of VR devices such as head-mounted displays, and the second is launching free versions of the most powerful engines (e.g., Unreal Engine^1^ and Unity^2^) (Checa & Bustillo, 2020). Thus, VR has become an alternative technology that is capable of being used in different areas, for example, in the treatment of autistic children.

### Related work

In recent years, some studies have been conducted using VR in the treatment of autism. One example of using VR in therapy is “Bob’s fish shop”, a game developed in the Unity game engine (Stewart Rosenfield et al., 2019). For interaction with a virtual environment, this project used the Oculus Rift headset and microphone for voice input, and the game was developed to improve the conversational etiquette of children with autism (Stewart Rosenfield et al., 2019).

Another project for rehabilitation used an IVRS that consists of L-shaped screens called “semi cave”, a robot with an eye-in-hand camera, and a sound system (Lorenzo et al., 2016). The IVRS uses a computer vision system to automatically determine children’s facial expressions during several social situations (Lorenzo et al., 2016). Here, a robot camera can fix the emotional state of the child and registers the number of times when the facial expressions of the child do not correspond with a given situation. Accordingly, this project allows children with ASD to train in new situations and tasks (Lorenzo et al., 2016).

Another example is the Cave Assisted Virtual Environment (CAVE), which provides a feasible solution for some children with ASD who have cognitive and sensory troubles, and who may not accept head-mounted displays. Interaction with virtual elements takes place without wearing additional devices (Alcañiz et al., 2022). By using the CAVE system, differences between autistic and typically developing children are found using eye gaze as a biomarker, and studies have shown that children with ASD spend less time looking at eyes, mouths, and faces than typically developing children during social situations (Alcañiz et al., 2022).

Moreover, the application of a Collaborative Virtual Environment (CVE) has also been seen to have positive effects on the rehabilitation of children with autism (Zhao et al., 2016). In this system, participants improve communication skills by playing collaborative games using hand movement that is tracked in real-time through cameras (Zhao et al., 2016). One of the advantages of this game is that people from different places can connect to the game using the internet, and interact with the CVE application through the Leap Motion controller.

One of the helpful projects for people with autism is called “My Automated Conversation Coach (MACH)”. The system provides training in social skills via virtual agents (Hoque et al., 2013). The virtual agent interviews the user, in addition to reading facial expressions and understanding the voice of the user, and is also able to answer the user in verbal and nonverbal manners (Hoque et al., 2013). In Table 1, we present a comparison between the related works.

**Table 1:** Comparison of related works

Study	(Ramachandiran et al., 2015)	(Stewart Rosenfield et al., 2019)	(Alcañiz et al., 2022)	(Zhao et al., 2016)
Country	**Malaysia**	**USA**	**Spain**	**USA**
Type of technology	VR and picture exchange communication system (PECS)	VRE and AR via Oculus Rift VR headset	Cave Assisted Virtual Environment (CAVE), machine learning and eye-tracking tools	Hand-in-Hand (HIH) Collaborative Virtual Reality (CVE) system, Leap Motion device
Number of participants	41	2	55	12
Results	From the indoor environments, the virtual toilet (66%,) was the most popular environment for behavioral training for autistic children. The classroom environment (46%) was the most required place as an outdoor environment for training.	The study was a positive experience for the participants. After finishing training, communication skills were immediately exercised in real life.	Children with ASD spend less time looking at eyes, mouths, and faces than typically developing children during social situations.	Participants with ASD showed comparably high interest in the games compared to typically developed children.

In summary, most of the related works used face recognition to identify the children’s behavior in the different situations designed in the virtual world. Moreover, such systems allow for providing psychological treatment for various anxiety disorders (Arthur et al., 2021). All of the mentioned studies are aimed at improving social interaction using VR. The main feature of the proposed study is the opportunity for the participants to train and improve their communication ability in an ecologically valid environment by practicing skills repeatedly, without leaving home. In previous works, in order to conduct training, it was necessary to install special equipment, such as a Cave Assisted Virtual Environment (CAVE), Leap Motion device, camera, microphone, computer, etc. The VR Oculus Quest2 system allows the user to walk around a virtual world for more active experiences, as well as access 360-degree visualization. In addition, Oculus Touch Controllers are built to deliver better gesture tracking, consequently making interaction with characters and objects of the VR possible. Finally, for carrying out therapy, parents just need the VR Oculus Quest2 and a smartphone. We also want to note that this is the first experience using the IVRS in Kazakhstan. This is especially important for Kazakh and Russian-speaking children, as such systems are only available in English. As far as we know, our project is the first to propose an IVRS for children with autism, that especially considers different patient aspects.

## Research design

The objective of this study is to present an IVRS to enhance the social interaction and communication skills of children with ASD. To evaluate the system, two test practices were conducted. In the first stage, testing was carried out at the university with typically developing children. Then, we tested the system in the Children’s correctional center “Intensive+” with autistic children. This is a private center located in Kazakhstan. Next, we present the characteristics of the system and present the conducted initial study.

### System features

**Fig. 1:**
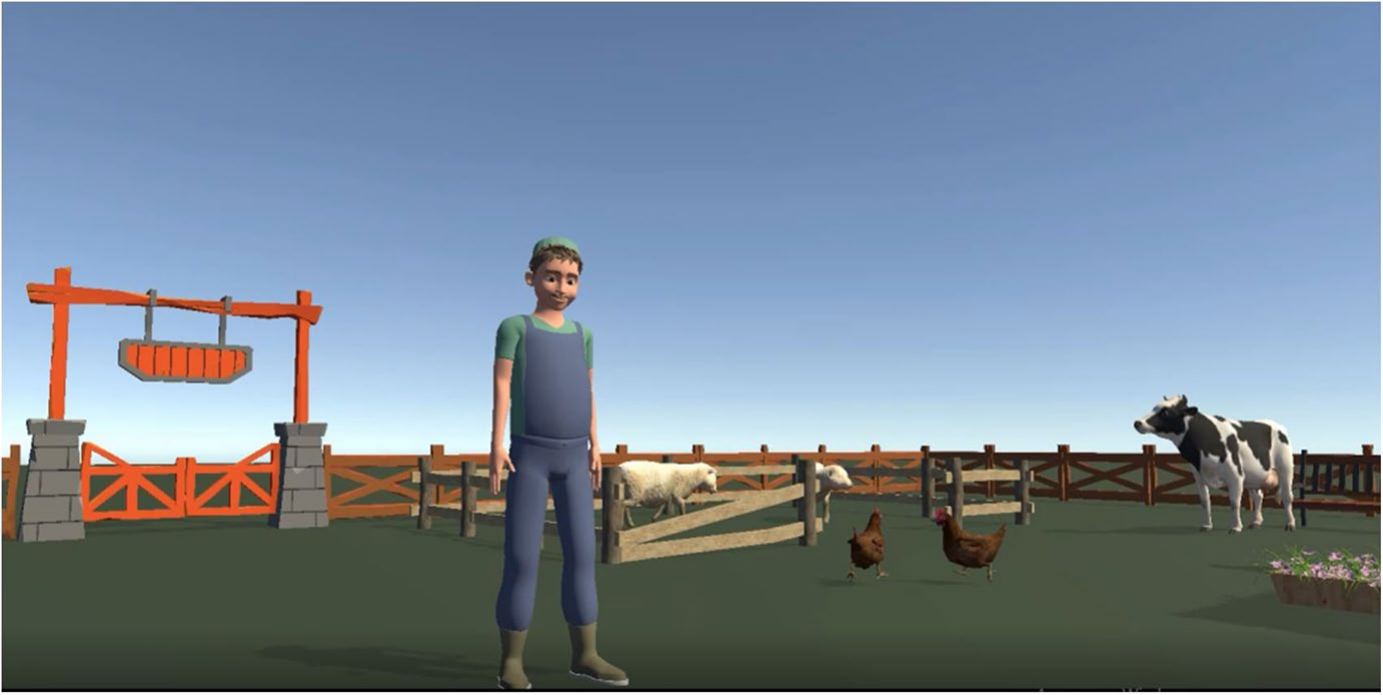
Meeting with the farmer

**Fig. 2:**
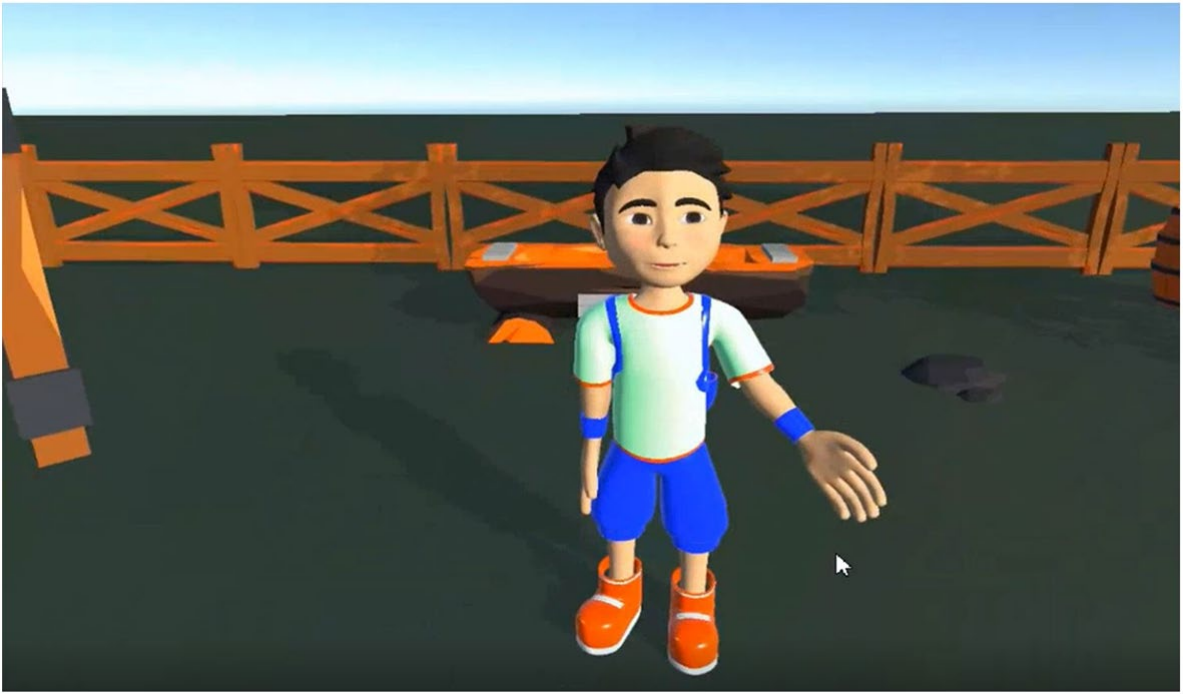
Communication with virtual boy

The system presented in this article is called “My Lovely Granny’s Farm”, and allows children to communicate with “the farmer”, ask questions, and get feedback. The virtual character asks the following questions: Hello, could you please wave me a hand? What is your name? Could you please tell me about yourself? After receiving a response from a participant, the virtual hero praises them for their answers. This project created a realistic 3D farm with animated domestic animals and birds, because earlier research works have demonstrated that “the human-animal bond can confer many health benefits for children with ASD” (The Human Animal Bond Research Institute (HABRI), 2020). The system aims to encourage children to continue further communication, and to feel comfortable themselves. We expect the VRTS to be beneficial not only to the patients, but also to offer a new experience for therapists as well. Figure 1 and Fig. 2 demonstrates the view from the VR glasses.

At first, an area in physical space needed to be set up using a wireless controller where the VR training takes place. The virtual therapy begins when the user wears a VR Oculus Quest2 headset. The gates to the farm open, and the user enters the farm. Users can walk through the farm to explore the surroundings. The farmer greets the guest and starts the farm tour to introduce the animals. The game’s main character (Mr. Farmer) changes his behavior depending on the emotions and actions of the participant. The farmer asks questions to enhance the communication skills of the participant. Also, users should carry out tasks. One of the important parts of the equipment is a sound system, which provides the virtual environment with sounds like in the real world.

**Fig. 3:**
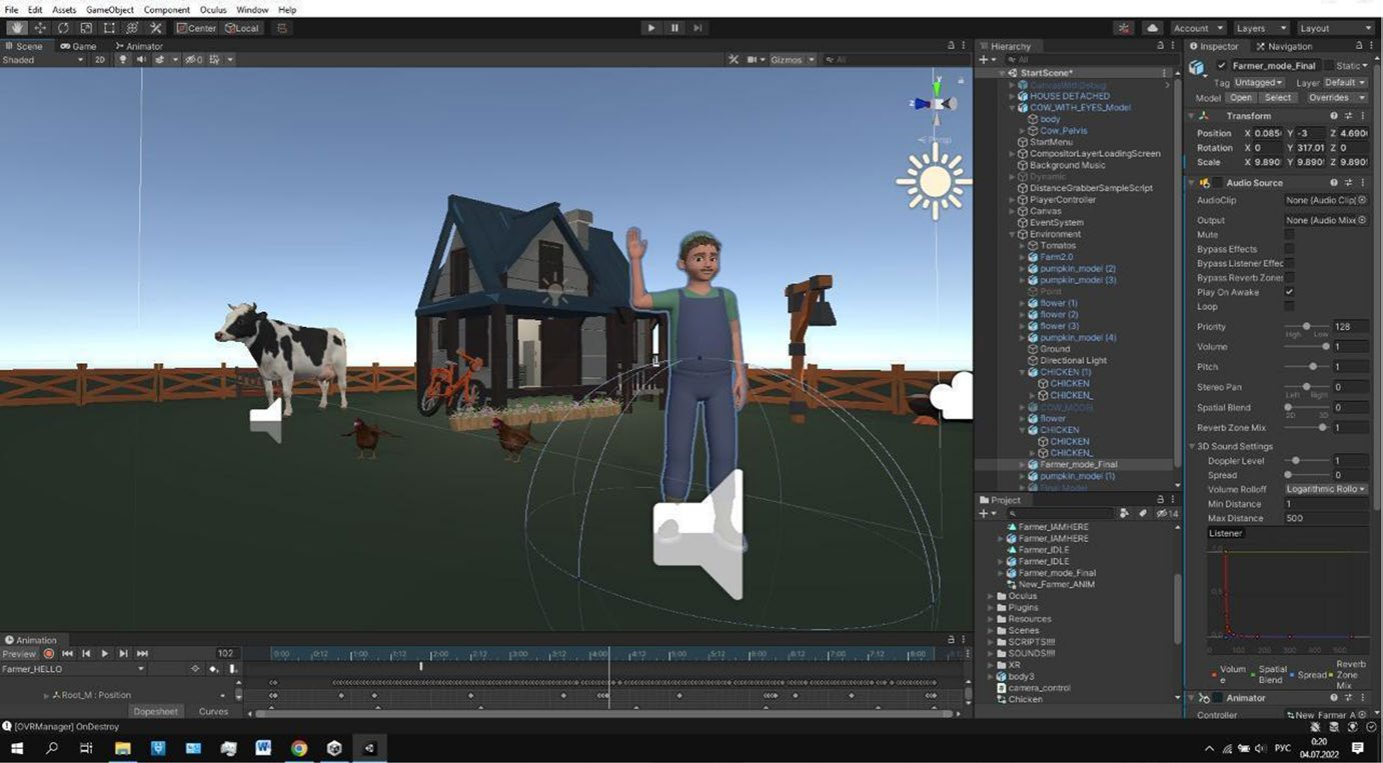
Greeting task

**Fig. 4:**
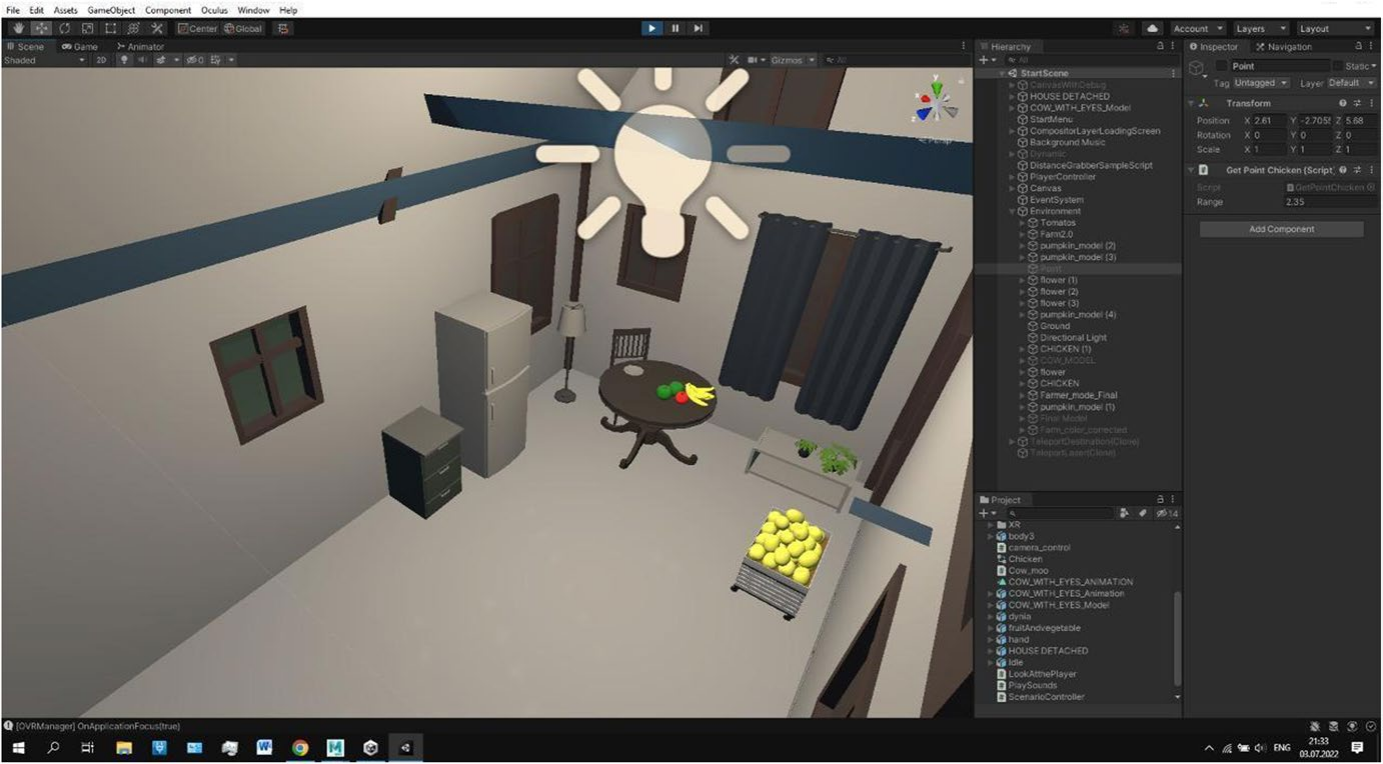
Entering home task

In Fig. 3, we demonstrate the virtual environment in the Unity software. In this scene, a virtual character greets the participant of the study. Figure 4 shows the interior of the farmer’s house. Participants should enter the home and interact with virtual objects.

- First task: Exploring the VR environment and greeting Mr. farmer

The teacher explains the situation and asks the participant the following questions: 1) Where are you? 2) What do you see? 3) Do you like this place? 4) What do you want to do? During the session, children receive support from the teacher to adapt to the environment. After adjusting to the VR farm, participants should greet Mr. Farmer and answer his questions.

- Second task: Entering the home, and interacting with Mr. farmer and the animals

The main aim of this study is to improve the social interaction of autistic children. Therefore, it is essential that the child communicates with the virtual character. The teacher gives an explanation of the social situation and recommends the following actions: 1) Do you want to enter the home? Please, try to enter and say what you see. 2) Do you hear sounds? 3) Do you hear mooing cows? Come closer to the cow, please. 4) Come closer to the farmer and say “Hello”.

#### Computational aspects

Virtual scenes and 3D characters were created in Autodesk Maya (n.d.)^3^ software. Autodesk Maya is a powerful software with tools for 3D modeling, animation, and rendering (Autodesk Inc., 2022). All of the created objects are exported in *.fbx format to the Unity game engine. Unity is a game engine that allows the development of mobile games and projects for PCs (Windows, iOS, Linux), and consoles such as Xbox, and Playstation (Unity documentation, 2021). It has various tools for working with graphics, animation, object physics, sound, templates, and scripts. In Unity, we use C# programming language for integration with the 3D environment, as well as face and sound recognition. Unity Face Recognition Emotion detection SDK enables the creation of scenarios where the user’s moods can be detected for the following base emotions: *i)* happy, *ii)* surprise, *iii)* angry, *iv)* sad, *v)* afraid, *vi)* disgust and *vii)* neutral (Unity documentation, 2021).

**Fig. 5:**
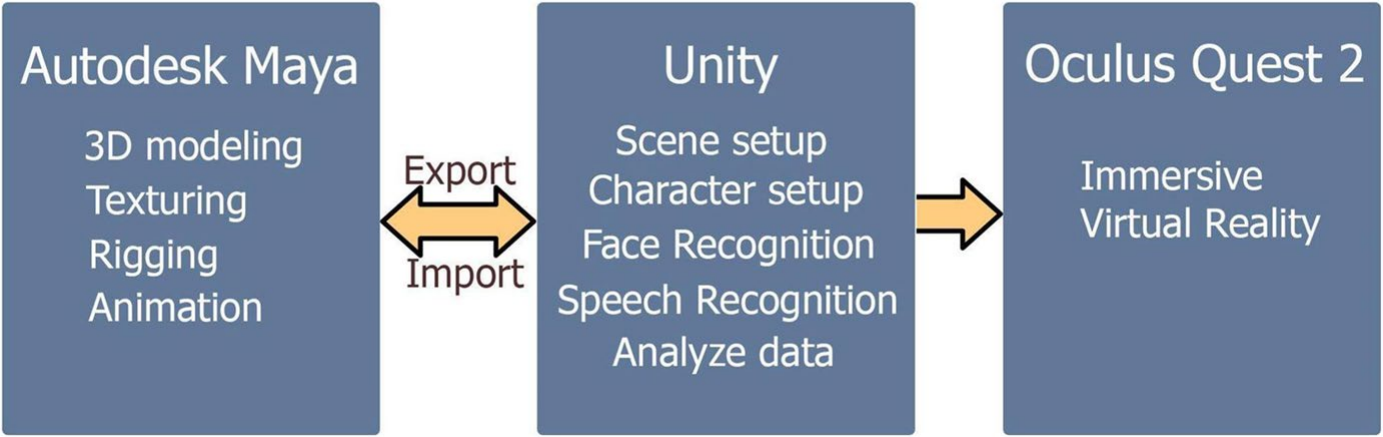
Project Block Diagram

**Fig. 6:**
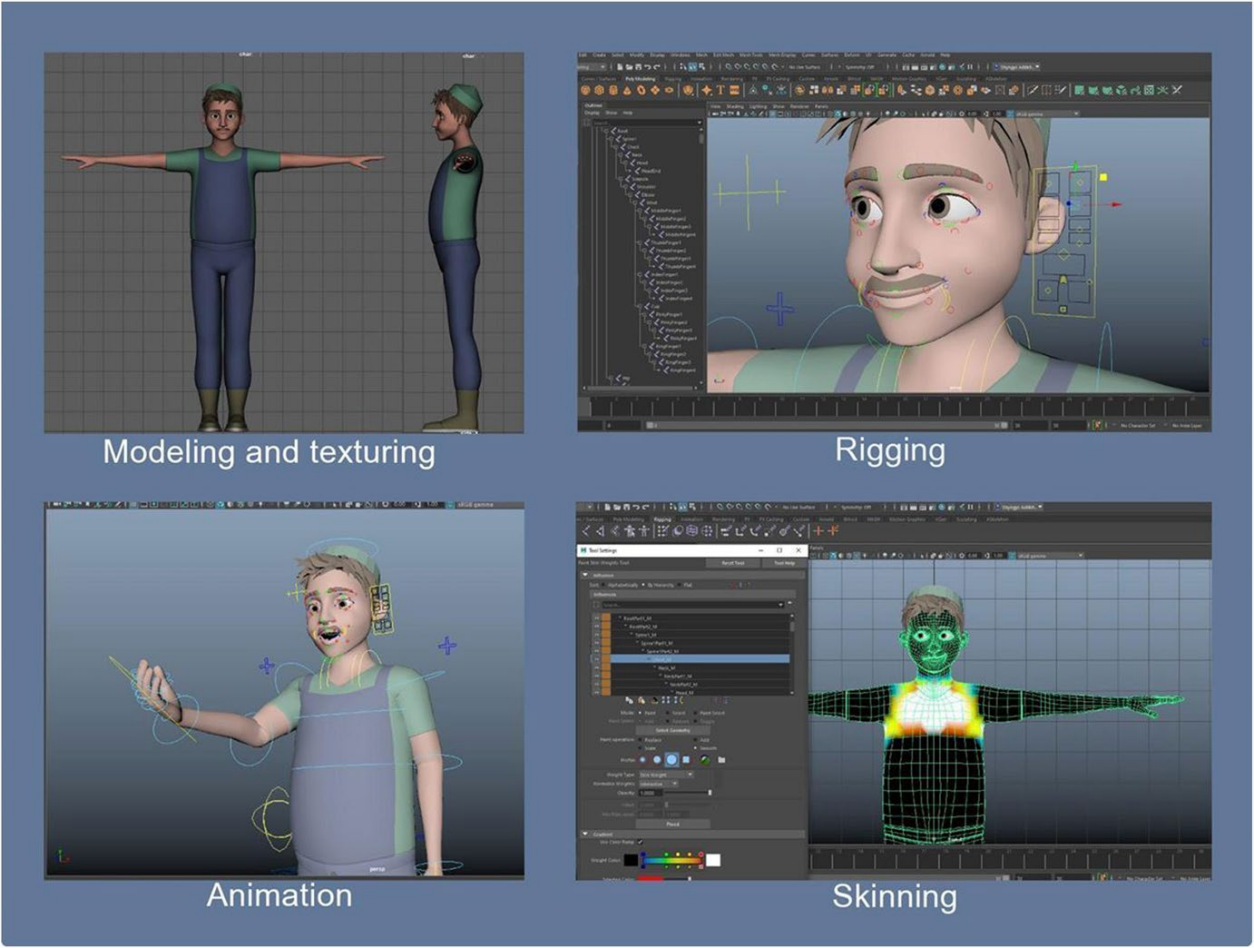
Work process in Autodesk Maya

Unity 2019.3 and newer versions use their own VR integration tool, XR SDK, which enables them to integrate with the Unity engine and fully use its features (Unity documentation, 2021). All processes of training have been recorded and shared with specialists in healthcare. In Fig. 5, we present a block diagram of the system described above. In Fig. 6, we demonstrate the work process carried out in Autodesk Maya (n.d.) software.

#### Educational aspects

There is no optimal method for the treatment of ASD that gives tangible results when working with patients of different ages (BRT neurorehabilitation center, 2022). One of the traditional treatments is behavioral therapy (Matspen, 2022). Because it is difficult to establish human-to-human interaction for children with autism, using IVRS is a good solution as an educational tool (Ramachandiran et al., 2015). The positive effect of the proposed therapy is based on the principle of a conditioned reflex. By helping a child develop the necessary skills, we reinforce each of their actions with a certain reward. For each completed task, participants gain scores. The system is composed of two main tasks aiming to enhance the communication skills of ASD children. In Table 2, we summarize these tasks.

**Table 2:** Description of the tasks

Id	Task	Duration	Description	Educational objective
1	Greeting	15 minutes	Walking and exploring the virtual farm. Participants should say the names and colors of the objects. Then come closer to the farmer and say hello.	Adaptation to an unfamiliar environment. Improving social skills.
2	Entering home	20 minutes	Exploring the interior of the house. Come closer to the plants, vegetables, and domestic animals. Determine the sound of the animal. Greet the farmer and state your name.	Getting to know new things. Overcome fear of new places.

In this system, participants could train social abilities in an explorative and safe environment with the support of the virtual character and specialists. One of the important elements of high influence on learning is the user’s interaction with the virtual environment. Unlike traditional teaching environments, using VR games for education and training is one of the best ways to achieve interactive learning (Checa & Bustillo, 2020).

### Materials and method

Interaction with the virtual world is provided by using the Oculus Quest2 wireless VR headset that tracks not only your head turns but also your position in space. The set includes two wireless controllers with touch areas on all buttons, triggers, and a joystick, from which the application understands the position of the hand in space and the position of the fingers on the controller (Facebook Inc., 2019). In order to prevent children from colliding with objects a spacious and empty room was used for the training. The training process is streamed on a laptop or smartphone, allowing us to monitor and record all of the participant’s actions. This gives us an opportunity to conduct observation research of the study.

#### Participants and data collection

The sample of our study was composed of 12 children (four girls and eight boys), aged between 4 and 15 with an ASD diagnosis, and 6 specialists from the center. The whole learning process was attended by two founders of the “Intensive+” rehabilitation center for children with autism, who are also specialists in child rehabilitation. Four specialized staff members were also present and accompanied their students. The specialists suggested starting training with group 4 participants (see Section 2 for a review of the group definitions), as only children in group 4 with autism attempt to engage in dialogue with their circumstances (active and verbal), although they have difficulty organizing it. The mental development of these children is more evenly delayed (Almanac Institute of special education, 2022). The “Intensive+” rehabilitation center was founded in 2021 by Kudaybergenova Gulzhan Kansarovna. She is the director of the center and works as a therapist with ASD children. In addition, she is a senior lecturer in the Department of Psychology of the University of Turan in Almaty city.

A memorandum of cooperation with the “Intensive+” center was obtained, together with a written agreement from parents that they did not object to their children participating in the training. Instructions on using equipment and the system principles were carefully explained to the participants.

**Fig. 7:**
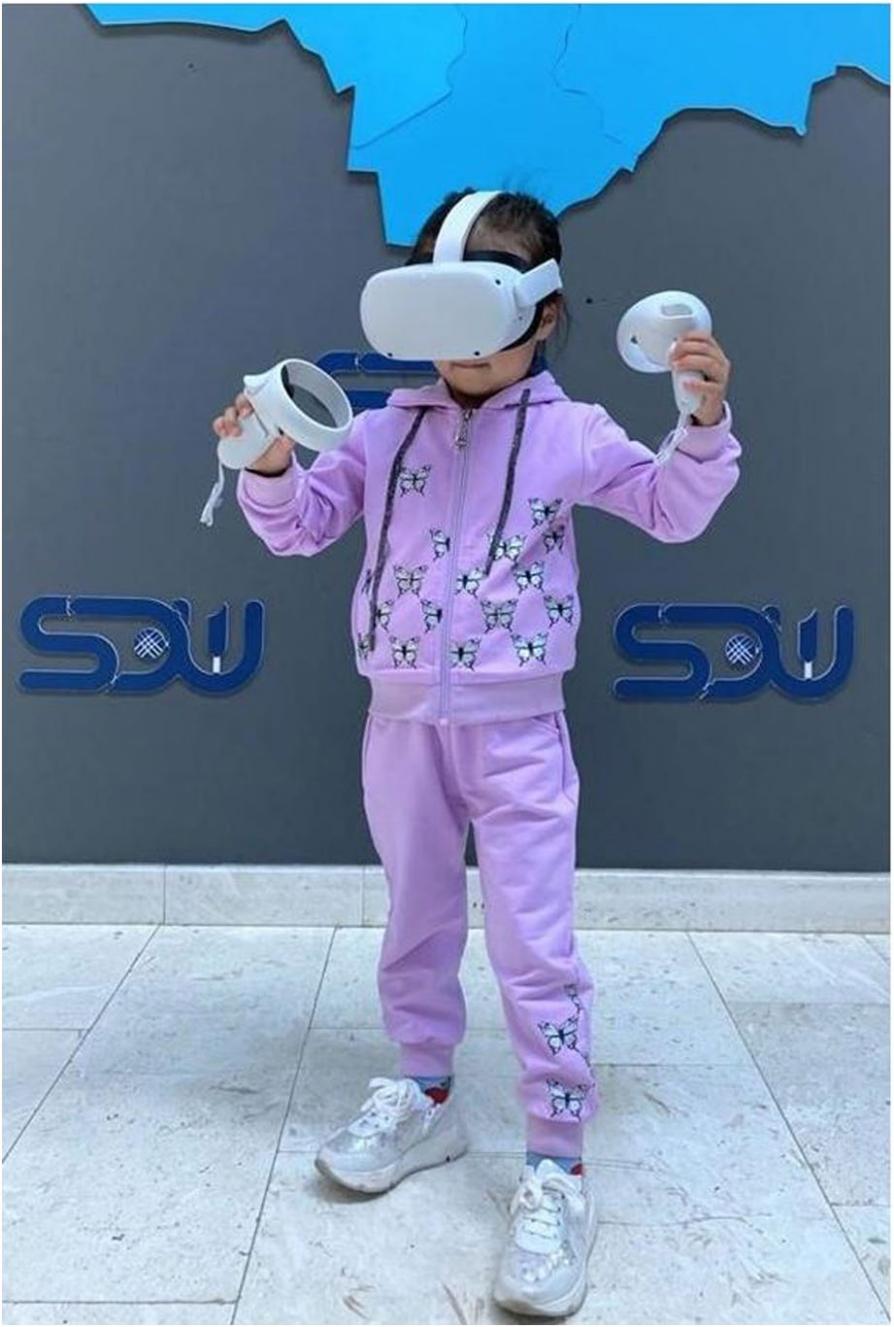
Testing the system with typically developing children

**Fig. 8:**
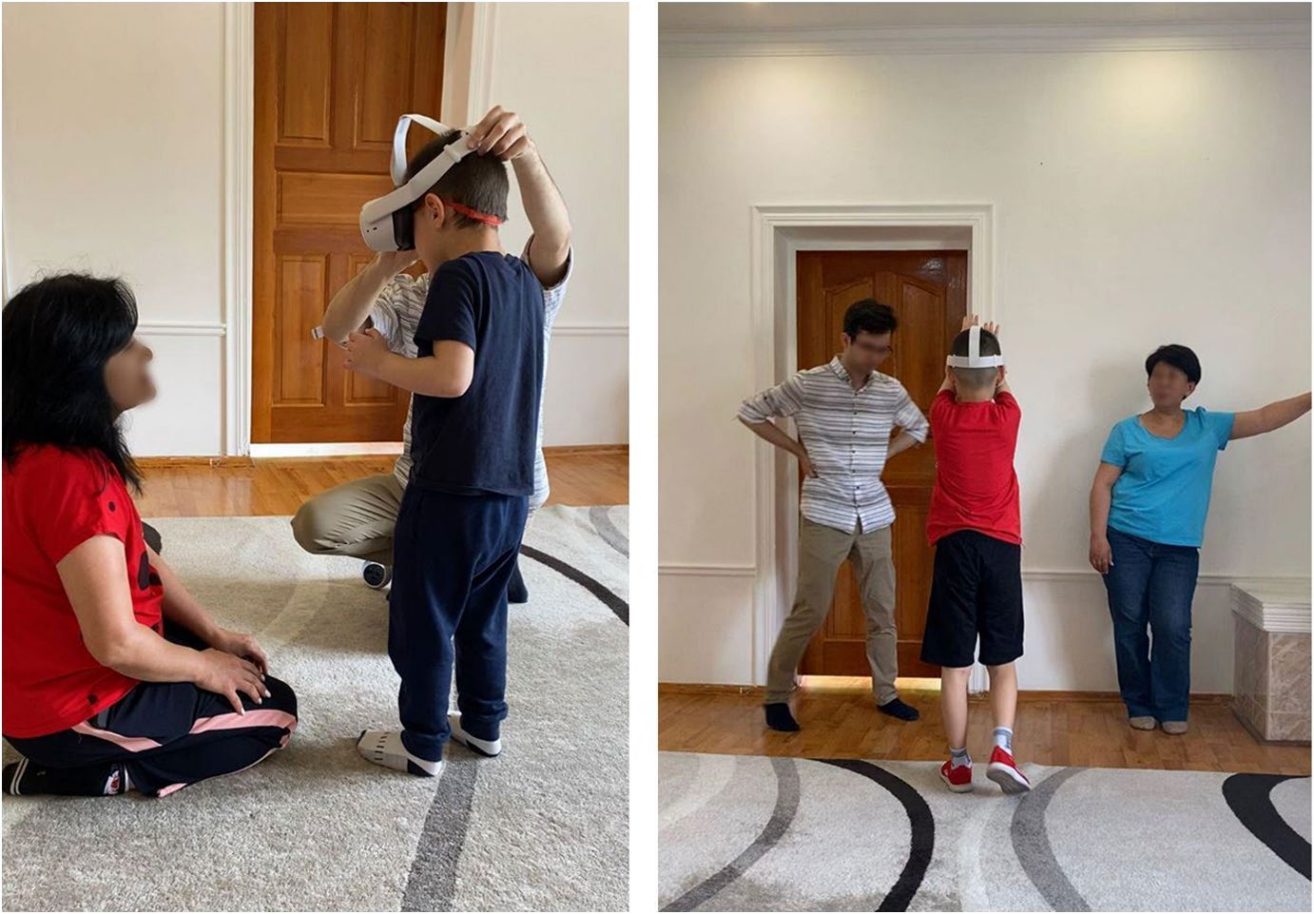
Training process with autistic children

Before starting the training, we explained to each child about the VR headset, and controllers, and how to use them. Specialists of the center and parents could watch the process of the training from the sidelines. After preparation, users wear the VR headset and immerse themselves into the virtual world. Figure 7 shows an example of testing the system with typically developing children in the university. Figure 8 shows the training process for autistic children carried out in the rehabilitation center.

In this study, we used qualitative analysis. Qualitative analysis was organized by asking questions to the participants during the training. In order to determine impressions from the training, we also questioned them after finishing the training process. The recorded video of the training was reviewed and analyzed by specialists. The participants immersed themselves in the Virtual Farm for approximately 15–20 minutes. Their answers to given questions, states of emotions, and carried-out task results were recorded.

## Results

Initially, we present the general results regarding the study. 10 participants wore VR headsets on the first attempt. The remaining two agreed to wear glasses on the second attempt. Most of the participating children (more than 8 children) were active during the therapy and tried to interact with a virtual character. Two participants tried to put on and take off the glasses several times because they wanted to compare the real world to the virtual. 2 children did not interact with the virtual character, and 1 child said that he was afraid of the farmer.

In order to improve the quality of the training, we asked several questions of the two main specialists about the diagnosis of children participating in training and how to improve our system. The specialists who analyzed the system presented recommendations and advice on how to better develop a virtual environment for training. Table 3 shows all of the given recommendations of the therapists from the rehabilitation center. After finishing the training, the participants were interviewed by the specialists in order to share their feelings and emotions about the game.

**Table 3:** Recommendations of the specialists from the rehabilitation center

**#**	Questions	Recommendation
1	Which autism group is the best to train with?	Starting training with the 4th group of autism
2	Which is the best way to build a virtual farm so that the participant feels comfortable and will not be intimidated?	Start training with one virtual character and gradually increase the number
3	Are there any specifics or rules for using the color palette in the Training system for ASD children?	Use autism-friendly color pallets in the Virtual environment - irritating colors (red, yellow, and white) can be slowly added to the scene, as children will have to face them in real life
4	What else to consider in a virtual scene?	It is important that the virtual scene does not contain sharp movements (flashes or fast animation) that may frighten the patient
5	Could you give some recommendations about animal animation in the scene?	Animals should not open their mouths, at the most they can lift their heads
6	Could you give some advice about the virtual characters of the training?	The Virtual farm and characters should be realistic

As a result of the study, we observed that children with autism are more focused on static objects and details. It should be noted that parents also showed their interest in using this system for therapy. Table 4 shows the characteristics of the participants and the results of the training. For every completed task, the participant scored 1 point. The results of the second task are slightly better than the first task. This indicates that the children began to adapt to the learning environment, thereby showing better results.

**Table 4:** Participants’ characteristics and results

Id	Age	Gender	Diagnosis	First task	Second task	Total
1	6	Male	ASD 4th group	5	5	10
2	10	Male	ASD 2-4th group	3	4	7
3	6	Male	ASD 2-4th group	0	2	2
4	7	Female	ASD 4th group	5	5	10
5	6	Female	ASD 4th group	5	5	10
6	5	Male	Delayed speech development with elements of autism	2	0	2
7	4	Male	ASD 1-2th group, anxiety dysfunction	0	0	0
8	6	Male	ASD 2-4th group	2	3	5
9	5	Male	ASD 2-4th group	3	5	8
10	15	Female	ASD 4th group	5	5	10
11	6	Male	mental delays, autism	4	5	9
12	5	Female	ASD 2nd group	0	1	1

Seven participants expressed curiosity about the Oculus Quest2 VR headset. The center specialists noted that both the children and their parents were looking forward to the next training, which is another indication that this study had a positive effect on children with ASD. From the results, we can see that the system is best directed to children with ASD from the 4th group. Girl with 2nd group of ASD screamed and resisted on her first attempt to put on VR glasses. Children from group 1 autism with anxiety disorder refused and were afraid of wearing the VR headset.

### Discussion

Autism is a developmental disorder that appears in the first three years of age in children (Autism Research Institute, 2021). Children with autism develop unusual fears that limit their ability to perform daily activities (Ramachandiran et al., 2015). Consequently, children with ASD need an efficient learning tool to advance their social skills (Ramachandiran et al., 2015). In this study, we proposed a virtual farm with domestic animals designed to enhance the behavioral and communication skills of autistic children. Considering the health conditions and age of the participants, using methods such as interviews or questionnaires is possibly quite problematic. Therefore, the best option is to use an observational research method. This system is an innovative solution for inclusive education for ensuring equal access to education for all students, taking into account the diversity of special educational needs and individual opportunities. Using this system, parents will be able to train without having to leave home.

The results of the study indicate that children with ASD mainly focused on static objects rather than active ones. The data contributes to a clearer understanding that children with autism show a deep interest in detail. Communication with virtual characters is an important part of their training because developing social skills is the first step in socialization. As such, we tried to pay special attention to the greeting task. During the training, two participants with typical language skills expressed a desire to play a game of “rock-paper-scissors” with a virtual boy. We performed their wish and saw a positive response to the interaction with the virtual boy. Animals are described as social catalysts with a positive effect on human interaction (McNicholas & Collis, 2006). The outcome of the study showed that the choice of the virtual farm as a scenario for the training was appropriate. 70% of the participants enjoyed watching the animals and tried to catch them. Researchers have found that animal-assisted interventions are convenient for improving social interaction and communication between people (Becker et al., 2017). The presence of the animals facilitated a smoother adaptation of the ASD children to a new environment. The children also tried to taste virtual fruit and wanted to ride a bicycle and drive a tractor.

Children of the fourth group of ASD with typical speech development more easily interacted with virtual characters than children with a speech impairment. Participants with speech disabilities mostly tried to ignore the farmer’s address and answered questions only when the teacher repeatedly asked the farmer questions. One of the major signs of autism is difficulty focusing on something they don’t interested (Syeda et al., 2017). In the first stages of the training, 50% of the participants just observes the Virtual world for a few minutes and refused to continue training as they had no interest in communicating with the farmer. After finishing the VR training, we tried to exercise conversational skills with ASD children in the real world. Additionally, specialists asked questions about impressions from the training.

Previous related works have compared results between typically developing and ASD children (Zhao et al., 2016; Alcañiz et al., 2022). In the presented study, we do not compare children with various developmental characteristics, and the study’s main objective was to develop social skills by providing repetitive training in a secure atmosphere.

Despite the voluminous literature review we did before starting to develop the system, we felt it necessary to ask questions of the specialists at the center for the rehabilitation of children with autism. As this was our first experience working with children, we had to prepare carefully for the training.

The specialists mentioned that we should train children with the 4th group of autism, which implies that it will be hard to provide training for children from the other 3 groups. The second specialist mentioned, “*Start training with one virtual character and gradually increase the number*”. Here, it can be held that ASD children need to adapt to the new environment and characters, and if they become afraid of the surrounding environment, this may lead to the rejection of further training.

The third specialist advised, “*Using autism-friendly color pallets in the Virtual environment. Irritating colors (red, yellow, and white) can be slowly added to the scene, as children will have to face them in real life*”. This corroborated the study conducted by (Grandgeorge & Masataka, 2016), and can help them to prepare for actual situations. This supports the idea that it is important to be concerned about system colors, as colors can directly affect the user’s experience.

One of the main recommendations of the specialist was that the virtual scene should not contain sharp movements, flash effects, loud sounds, and fast animation. For example, if all the farm animals make noises and quick movements at the same time, it may frighten the child. According to Kanner (1943), children with autism have atypical fears of specific loud noises and moving objects. Following this recommendation, we avoid including these types of features in the system.

Another piece of advice was “*Animals should not open their mouths; at the most they can lift their heads*”. As mentioned above, ASD children are afraid of some actions and effects. That is why we tried to make smooth animations of the animals. The last recommendation was that the Virtual farm objects and characters should be realistic. To make the training as effective as possible, we have tried to make the participants feel like they are in the real world.

In creating a virtual environment, we took into account all of the recommendations of experts, as well as the experiences drawn from previous research. Compared to other studies that require expensive and space-consuming implementations, our system is more mobile, and for the purposes of therapy, it needed just a VR Oculus Quest2 headset and a computer.

According to Table 4, children showed comparatively good results during the second phase of training, and children who refused to wear VR headsets on the first attempt agreed to put them on the second time. The training results show that 30% of the children had difficulties with the “Greeting task” with the farmer. Specifically, they needed more time for responding to the greeting, and with the support of the specialists at the center, the children finally tended to answer him. For 95% of the girls, it was easier for them to complete the tasks, and during the “Entering home” task, one 15-year-old girl with autism said that she would like to have a home like the one on the Virtual farm because she needs personal space. Overall, in their interaction with the VR environment, all of the children focused their attention on static objects and natural elements. Following the results of our study, participants showed a good interest in IVRS. We also tried to use the learned skills immediately after finishing the VR training. This result is similar to the study conducted by Stewart Rosenfield et al., called “A Virtual Reality System for Practicing Conversation Skills for Children with Autism” (Stewart Rosenfield et al., 2019).

### Limitations

Our study has some limitations that we seek to mitigate and report, so as to avoid any possible misunderstandings regarding the study, as well as to facilitate the evolution of the research in future studies. Initially, the number of participants may not be adequate to yield deeper and more generalizable results. It is also possible to note that the participants had different ages and different levels of autism, which can decrease the generalizability of the results. To mitigate this limitation, we used data analysis techniques widely used and merged in studies of this type, and in both cases, we recommend that future studies be conducted with larger samples.

The fact that all participants are from the same country may also indicate that the results may change according to the demographic conditions of the participants. However, these limitations make room for the study to be replicated in different countries. Regarding the technical limitations of the study, the virtual reality headset may not be accepted by all children, given that ASD children have different sensory issues (Autism Speaks, 2021; Pandey & Vaughn, 2021). Another possible limitation is that some parents who only believe in traditional medicine, might not permit therapy with a VRTS. However, there was no incidence of this being an issue in the present study.

### Observed impact

We developed this system in such a way that determines what the child pays attention to during the training. It is also important to determine how much time is needed for a child to adapt to the new environment, and to start to respond to the virtual character. The VR Oculus Quest 2 headset has built-in video capture capabilities and transfers recorded videos to other devices. This feature allows for recording the entire learning process and an analysis of the collected data. The data can then be reviewed by therapists and parents to determine the success and mistakes of the children. Overall, the developed system has shown its positive potential for children with autism in the 4th group. However, a study of a large number of children with ASD is needed to confirm the effectiveness of the system.

## Concluding remarks

In this study, we proposed an innovative solution (i.e., a VR Training system) for rehabilitating children with ASD. This system may be offered to users with a high degree of interactivity, and allows them to practice various social situations in a safe and controlled environment. To use this training system a person just needs a VR Headset and an application on a smartphone. In conditions such as those of the Covid-19 pandemic, this training system is especially relevant, although people from remote areas can also take the opportunity to continue therapy in their own homes. The VR Oculus Quest2 headset allows multiple connections by different users to the training system. Psychotherapists can also carry out treatment by connecting to the system as a virtual character. We conducted a study with 12 participants and identified that children with autism explore the virtual environment with high interest, and mainly focus their attention on details and static objects. In future studies, we aim to include face and speech recognition in our system and to replicate the study with a larger sample size and in different countries.

## Footnotes

1 © 2004–2022, Epic Games, Inc. https://www.unrealengine.com/en-US

2 © 2022 Unity Technologies. https://unity.com/ru

3 © 2022 Autodesk Inc. https://www.autodesk.com/products/maya

## References

[CR1] Alcañiz M, Chicchi-Giglioli IA, Carrasco-Ribelles LA, Marín-Morales J, Minissi ME, Teruel-García G, Sirera M, Abad L (2022). Eye gaze as a biomarker in the recognition of autism spectrum disorder using virtual reality and machine learning: A proof of concept for diagnosis. Autism research: official journal of the International Society for Autism Research.

[CR2] Alimanova, M., Soltiyeva, A., Urmanov, M., & Adilkhan, S. (2022). Developing an immersive virtual reality training system to enrich social interaction and communication skills for children with autism Spectrum disorder. *2022 international conference on smart information systems and Technologies (SIST)*. 10.1109/sist54437.2022.9945733

[CR3] Arthur, T., Harris, D., Brosnan, M., Wilson, M., Williams, G. K. R., & Vine, S. J. (2021). An examination of active inference in autistic adults using immersive virtual reality. *Scientific Reports, 11*(1). 10.1038/s41598-021-99864-y 10.1038/s41598-021-99864-y PMC8514518 34645899

[CR4] Autism Research Institute, 2021, accessed 20 October 2021 www.autism.org.

[CR5] Autism Speaks, 2021, accessed 15 September 2021, https://www.autismspeaks.org/autism-statistics-asd.

[CR6] Bal VH, Wilkinson E, Fok M (2022). Cognitive profiles of children with autism spectrum disorder with parent-reported extraordinary talents and personal strengths. Autism.

[CR7] Becker JL, Rogers ER, Burrows B (2017). Animal-assisted social skills training for children with autism Spectrum disorders. Anthrozoos.

[CR8] BRT neurorehabilitation center, 2022, accessed 20 May 2022, https://www.brtclinic.ru/lechenie/autizm/.

[CR9] Checa D, Bustillo A (2020). A review of immersive virtual reality serious games to enhance learning and training. Multimedia Tools and Applications.

[CR10] Finkelstein, S., Barnes, T., Wartell, Z., & Suma, E. A. (2013). Evaluation of the exertion and motivation factors of a virtual reality exercise game for children with autism. *IEEE Virtual Reality Conference*. 10.1109/vaat.2013.6786186

[CR11] Forbes Kazakhstan, 2021, accessed 11 September 2021, https://forbes.kz/process/education/v_kazahstane_deti_s_autizmom_poluchili_vozmojnost_hodit_v_shkolu.

[CR12] Grandgeorge, M., & Masataka, N. (2016). Atypical color preference in children with autism spectrum disorder. *Frontiers in Psychology, 7*. 10.3389/fpsyg.2016.01976 10.3389/fpsyg.2016.01976 PMC5179595 28066297

[CR13] HABRI. (2020). *The Human-Animal Bond for Autism Spectrum Disorder*. HABRI https://habri.org/blog/the-human-animal-bond-for-autism-spectrum-disorder/. Accessed 21 Apr 2023.

[CR14] Halabi O, El-Seoud SA, Alja’am, J. M., Alpona, H., Al-Hemadi, M., & Al-Hassan, D. (2017). Design of Immersive Virtual Reality System to improve communication skills in individuals with autism. International Journal of Emerging Technologies in Learning (Ijet).

[CR15] Hocking, D. R., Ardalan, A., Abu-Rayya, H. M., Farhat, H., Andoni, A., Lenroot, R. K., & Kachnowski, S. (2022). Feasibility of a virtual reality-based exercise intervention and low-cost motion tracking method for estimation of motor proficiency in youth with autism spectrum disorder. *Journal of Neuroengineering and Rehabilitation, 19*(1). 10.1186/s12984-021-00978-1 10.1186/s12984-021-00978-1 PMC8742363 34996473

[CR16] Hoque, M., Courgeon, M., Martin, J.-C., Mutlu, B., & Picard, R. W. (2013). MACH: My Automated Conversation Coach. *Proceedings of the 2013 ACM international joint conference on pervasive and ubiquitous computing*, 697–706. Presented at the Zurich, Switzerland.. 10.1145/2493432.2493502

[CR17] Никольская ОСН (2014). *Психологическая классификация детского аутизма*.

[CR18] Justham, L., West, A. A., Harrison, R. W., Jones, R. B., Harland, A. R., Caine, M. P., & Roberts, J. (2004). The use of virtual reality and automatic training devices in sport. *A Review of Technology Within Cricket and Related Disciplines*. 10.1115/esda2004-58257

[CR19] Kanner L (1943). Autistic disturbances of affective contact. The Nervous Child.

[CR20] Lele A (2013). Virtual reality and its military utility. Journal of Ambient Intelligence and Humanized Computing.

[CR21] Lorenzo G, Carreres AL, Pomares J, Roig RM (2016). Design and application of an immersive virtual reality system to enhance emotional skills for children with autism spectrum disorders. Computers & Education.

[CR22] Matspen (2022). *Israeli Private Center For Neurology, Psychotherapy And Psychiatry*.

[CR23] Maya Software *Get Prices & Buy Official Maya 2024 | Autodesk*. (n.d.). https://www.autodesk.com/products/maya. Accessed 21 Apr 2023.

[CR24] McNicholas J, Collis GM, Fine AH (2006). Animals as social supports. *Handbook on animalassisted therapy: Theoretical foundations for guidelines and practice*.

[CR25] Mikropoulos TA, Natsis A (2011). Educational virtual environments: A ten-year review of empirical research (1999–2009). Computers & Education.

[CR26] Miles HC, Pop SR, Watt SJ, Lawrence GP, John NW (2012). A review of virtual environments for training in ball sports. Computers & Graphics.

[CR27] Miller IT, Wiederhold BK, Miller CS, Wiederhold MD (2020). Virtual reality air travel training with children on the autism spectrum: A preliminary report. Cyberpsychology, Behavior and Social Networking.

[CR28] Pallavicini F, Argenton L, Toniazzi N, Aceti L, Mantovani F (2016). Virtual reality applications for stress management training in the military. Aerospace Medicine and Human Performance.

[CR29] Pandey V, Vaughn L (2021). The potential of virtual reality in social skills training for autism: Bridging the gap between research and adoption of virtual reality in occupational therapy practice. *The open journal of occupational*. Therapy.

[CR30] Park C, Yelland GW, Taffe JR, Gray KM (2012). Brief report: The relationship between language skills, adaptive behavior, and emotional and behavior problems in pre-schoolers with autism. Journal of Autism and Developmental Disorders.

[CR31] Radianti J, Majchrzak TA, Fromm J, Wohlgenannt I (2020). A systematic review of immersive virtual reality applications for higher education: Design elements, lessons learned, and research agenda. Computers & Education.

[CR32] Rahmadiva, M., Arifin, A., Fatoni, M. H., Halimah Baki, S., & Watanabe, T. (2019). A design of multipurpose virtual reality game for children with autism spectrum disorder. *2019 international biomedical instrumentation and technology conference (IBITeC)*. 10.1109/ibitec46597.2019.9091713

[CR33] Ramachandiran, C. R., Jomhari, N., Thiyagaraja, S., & Mahmud, M. M. (2015). Virtual reality based behavioural learning for autistic children. *Research Gate* https://www.researchgate.net/publication/283521739_Virtual_reality_based_behavioural_learning_for_autistic_children. Accessed 21 Apr 2023.

[CR34] Rosenfield NS, Lamkin K, Re J, Day K, Boyd L, Linstead E (2019). A virtual reality system for practicing conversation skills for children with autism. Multimodal Technologies and Interaction.

[CR35] Ruikar, D. D., Hegadi, R. S., & Santosh, K. C. (2018). A systematic review on orthopedic simulators for psycho-motor skill and surgical procedure training. *Journal of Medical Systems, 42*(9). 10.1007/s10916-018-1019-1 10.1007/s10916-018-1019-1 30073548

[CR36] Syeda UH, Zafar Z, Islam ZZ, Tazwar SM, Rasna MJ, Kise K, Ahad AR (2017). Visual face scanning and emotion perception analysis between autistic and typically developing children. International Symposium on Wearable Computers.

[CR37] Тевелев ДВ (2022). *ABA-терапия в лечении аутизма у детей*.

[CR38] Zhao, H., Swanson, A., Weitlauf, A. S., Warren, Z., & Sarkar, N. (2016). A novel collaborative virtual reality game for children with ASD to Foster social interaction. *Springer EBooks, 276–288*. 10.1007/978-3-319-40238-3_27

